# RENAL‐CHIP: Rejection Evaluation via Non‐Invasive Analysis of Circulating Podocytes With Herringbone‐Chip Isolation Platform

**DOI:** 10.1002/advs.202520426

**Published:** 2026-03-18

**Authors:** Juan Song, Yilong Liu, Zeyuan Zheng, Ming Zhu, Yuning Zou, Pei Liu, Yue Zheng, Xiyuan Yu, Jianzhong Zheng, Chen Shao, Huimin Zhang, Chaoyong Yang

**Affiliations:** ^1^ Discipline of Intelligent Instrument and Equipment College of Chemistry and Chemical Engineering Department of Urology Xiang'an Hospital of Xiamen University School of Medicine, Innovation Laboratory for Sciences and Technologies of Energy Materials of Fujian Province (IKKEM) Xiamen University Xiamen China; ^2^ Key Laboratory of Pathogenic Fungi and Mycotoxins of Fujian Province The Ministry of Education Key Laboratory of Biopesticide and Chemical Biology College of Life Sciences Fujian Agriculture and Forestry University Fuzhou China

**Keywords:** circulating podocytes, immune rejection, microfluidic interface, non‐invasive diagnosis, renal transplant

## Abstract

Immune rejection limits long‐term renal allograft survival, yet current diagnostics lack non‐invasive, and precise detection. Here, donor‐derived circulating podocytes (CPCs) are identified as a blood‐based surrogate of acute rejection. A magnetically reversible herringbone microfluidic chip equipped with epithelial cell adhesion molecule (EpCAM) antibody‐functionalized magnetic beads efficiently isolates CPCs from 1 mL of peripheral blood (capture efficiency 84.4%, release 95.5%, viability 96.0%). Among 65 participants, only transplant recipients with biopsy‐confirmed rejection exhibited≥35 CPCs mL^−^^1^ (AUC = 1.0). Fluorescence in situ hybridization (FISH) of sex‐mismatched transplants confirmed donor origin. Single‐cell RNA‐seq of 10 isolated CPCs revealed up‐regulation of podocyte‐injury and innate/adaptive immune genes (such as NF‐κB, TNF, NOD‐like receptor pathways) in rejection versus stable samples. CPC enumeration thus provides a minimally invasive, mechanistically informed warning of renal allograft rejection.

## Graphical abstract

RENAL‐CHIP converts 1 mL of peripheral blood into a biopsy‐equivalent readout of renal‐allograft fate. By magnetic capture and release of donor‐derived circulating podocytes through a herringbone microfluidic chip, 84% capture, 96% release and single‐cell RNA evidence of rejection‐specific immunity are achieved. The assay obviates invasive biopsy, enables immediate post‐transplant surveillance, and provides a scalable platform for personalized immunosuppression.

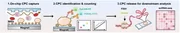

## 1 Introduction

Renal transplantation serves as the most effective treatment method for end‐stage renal disease, significantly improving patients' life quality and survival rates [1]. However, immune rejection remains a major barrier to long‐term survival in renal allograft recipients [2]. Although modern immunosuppressive therapy has reduced the incidence of rejection to some extent, about 10%∼15% of patients still suffer from immune rejection within the first year after transplantation [3, 4]. Currently, diagnosis of renal transplant rejection relies mainly on clinical indicators such as renal biopsy, serum creatinine levels, and urinary exfoliated cells. Among them, renal biopsy is the gold standard for diagnosing immune rejection, but it is invasive and can cause graft injury, infection, and patient discomfort [5]. Moreover, the limited sample size of biopsies and sampling errors may cause an inaccurate diagnosis. The serum creatinine level is important for monitoring renal function, but its specificity and sensitivity are inadequate, because of interference by non‐renal factors such as inflammation [2]. Urinary exfoliated cells are also limited in clinical application due to the challenges of sparse cell numbers, complex origins, biological variability, and interfering factors [6].

In recent years, liquid biopsy technology, which detects circulating biomarkers in body fluids such as peripheral blood, has been widely used in the early diagnosis of tumors, prenatal diagnosis, monitoring of cardiovascular diseases, and so forth [7, 8, 9]. This technology has the advantages of being non‐invasive, highly repeatable, and capable of providing real‐time monitoring, thereby greatly improving the efficiency of early disease diagnosis and treatment monitoring. In the field of renal transplantation, donor‐derived cell‐free DNA (dd‐cfDNA) has been proven to be a non‐invasive biomarker with potential for detecting transplant rejection [10]. Studies have shown that elevated levels of dd‐cfDNA often indicate ongoing inflammation and tissue damage in the transplanted kidney [11, 12, 13]. Unfortunately, dd‐cfDNA still faces some challenges in clinical application, such as low concentration, high fragmentation, and complex extraction processes [14]. In addition, dd‐cfDNA quantitative detection methods based on single nucleotide polymorphisms (SNPs) are subject to limitations, including false negatives and the impact of non‐specific factors, such as infections and inflammation [15, 16]. Therefore, identifying novel biomarkers for the diagnosis and prediction of renal transplant rejection is an important clinical issue that needs to be urgently addressed.

Podocytes are epithelial cells covering the outer side of the glomerular basement membrane, and their unique morphology and function play key roles in maintaining the integrity of the glomerular filtration barrier [17]. Moreover, podocytes can serve as important targets of immune responses, and they exhibit characteristics similar to immune cells, engaging in both innate and adaptive immunity [18]. A variety of factors, including mechanical, oxidative, or immune stress, can induce cell injury, eventually leading to cell detachment and the occurrence of proteinuria [19]. Upon injury, podocytes undergo dedifferentiation and re‐express epithelial markers such as cytokeratin [20]. We clinically observed epithelial cell adhesion molecule (EpCAM)‐positive podocytes in kidney injury tissues (Figure S1). Given that PODXL is predominantly expressed in podocytes and renal endothelial cells, while EpCAM is primarily expressed in epithelial cells, we classify PODXL+/EpCAM‐ cells as normal podocytes, PODXL‐/EpCAM+ cells as normal epithelial cells (e.g., renal tubular epithelial cells), and PODXL+/EpCAM+ cells as dedifferentiated abnormal podocytes. Inspired by circulating cell‐based liquid biopsy technology, we hypothesize that these podocytes are shed into the peripheral blood following immune rejection, forming circulating podocytes (CPCs) that may serve as an independent predictor of transplant rejection. Compared to urine, the whole blood exhibits minimal background interference from EpCAM‐positive normal epithelial cells and maintains more stable osmotic pressure and pH, which are conducive to preserving cell viability. To verify this, we designed a magnetically reversible microfluidic interface, which loads EpCAM antibody‐functionalized magnetic beads on the surface of a herringbone chip to generate turbulence and capture epithelial‐derived cells in patients' peripheral blood. EpCAM, podocyte‐specific marker (Podocalyxin, PODXL), and leukocyte‐specific marker (CD45) are used to identify the captured cells in the chip. Cells that were positive for EpCAM and PODXL but negative for CD45 are identified as CPCs. After removal of the magnetic field to recover the cells from the chip, fluorescence in situ hybridization (FISH) analysis on the CPCs from five renal allograft recipients proved that the CPCs originated from the renal transplant donor. Finally, comparison of the gene expression differences of CPCs between patients with and without immune rejection using single‐cell RNA sequencing (scRNA‐seq) elucidated the role of immunity in renal transplant rejection and the molecular mechanisms underlying the increased levels of CPCs in patients with immune rejection.

## 2 Results and Discussion

### 2.1 Working Principle of RENAL‐Chip

**FIGURE 1:**
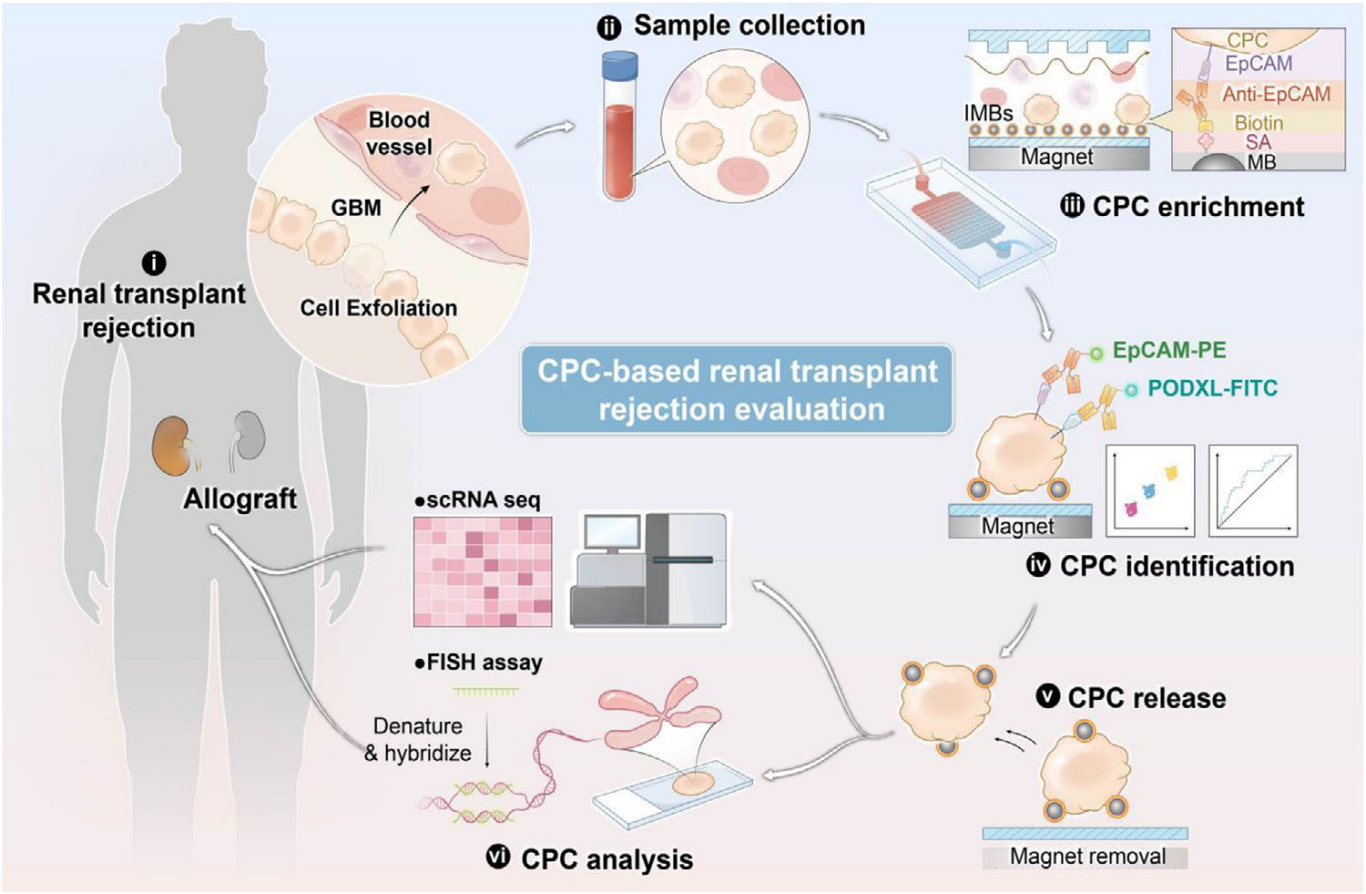
Overview of magnetic reversible microfluidic interface (RENAL‐Chip)‐based CPCs analysis for the diagnosis of renal transplant immune rejection.

Podocytes are terminally differentiated glomerular epithelial cells that detach when allo‐immune injury disrupts their cytoskeletal anchor. Once in the circulation, these fragments (CPCs) carry donor DNA and rejection‐specific transcriptomes. We therefore reasoned that a blood draw could replace invasive biopsy only if three stringent conditions were simultaneously satisfied: (i) specific enrichment—unambiguous isolation of PODXL+/CD45‐ podocytes against a 10^6^‐fold excess of leukocytes; (ii) high‐sensitivity capture—recovery of scarce CPCs (≤ 100 CPCs mL^−^^1^ whole blood) from 1 mL of blood; and (iii) reversible, gentle release—elution of live, intact cells compatible with downstream analysis. To meet these criteria, we engineered RENAL‐Chip, a magnetic–herringbone chip (HB‐Chip) for noninvasive capture and analysis of CPCs, whose chamber measures 40.0 mm in length, 8.4 mm in width, and 100 µm in height, with a groove spacing of 200 µm and a groove width of 100 µm (Figure S2). The complete operational workflow is schematically depicted in Figure 1. Briefly, a high‐affinity anti‐EpCAM layer is grafted onto 2.8 µm super‐paramagnetic beads. When the external field (4.78 mT) is applied perpendicular to flow, beads self‐assemble into 3D chains within the 100‐µm channel, raising the local antibody surface density to ∼ 2.03 × 10^3^ µm^−2^. PODXL+/CD45‐ podocytes are immobilized within seconds, whereas unbound leukocytes remain in the free stream, giving a high signal‐to‐noise ratio. To ensure high sensitivity capture, the herringbone floor was adopted to generate staggered Dean vortices. These vortices repeatedly sweep rare cells from the core to the magnetized interface, increasing the collision probability compared with a smooth channel. For gentle recovery of CPCs, the magnet is removed, and 5% BSA solution is injected into the chip to achieve non‐destructive cell release.

### 2.2 CPC Capture and Release Performance of RENAL‐Chip

**FIGURE 2:**
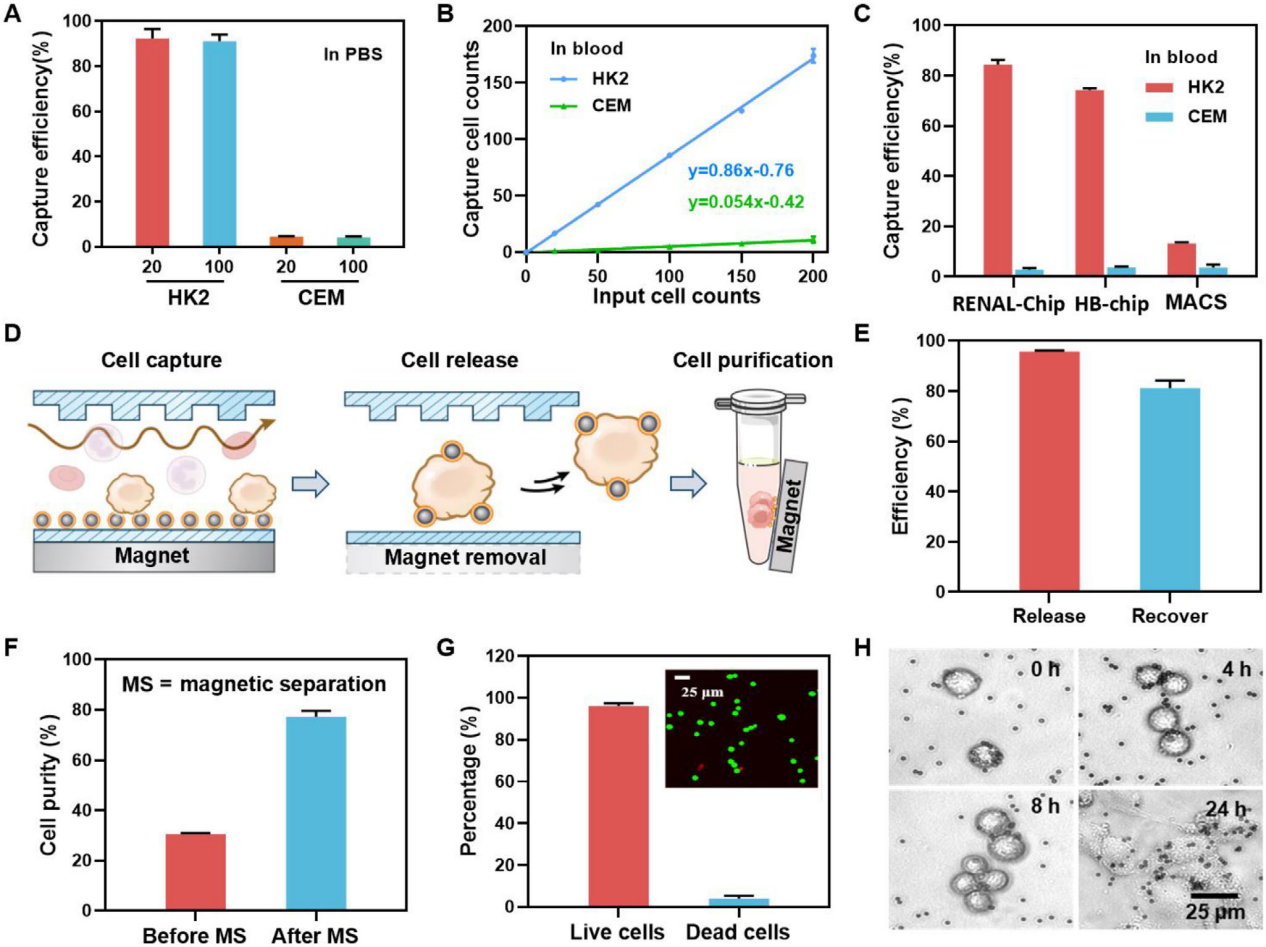
Performance of CPC capture and release of RENAL‐Chip. (A) Cell capture efficiencies of the chip in buffer. (B) Linear fitting of captured cell counts versus input cell counts in blood using the RENAL‐Chip. (C) Comparison of capture efficiency among different methods. (D) Schematic illustration of the cell capture and release process. (E) Release efficiency and recovery efficiency of HK2 cells. (F) Purity of HK2 cells before and after magnetic separation. (G) Cell viability of released HK2 cells. The inset shows a typical fluorescence image of released cells, with green dots indicating live cells and red dots indicating dead cells, scale bar = 25 µm. (H) Representative images of released HK2 cells cultured at different time points (0, 4, 8, and 24 h), scale bar = 25 µm. Plots and error bars represent the mean ± SD from three independent replicates.

Given that CPCs are epithelial‐derived cells expressing the EpCAM, we chose HK2 cells (human renal cell line) as the EpCAM‐positive cell line and CCRF‐CEM cells (human acute lymphoblastic leukemia T lymphocytes) as the EpCAM‐negative cell line. Flow cytometric analysis validated their EpCAM antigen expression levels (Figure S3). To optimize cell capture efficiency (CCE), we evaluated the dosage of immunomagnetic beads (IMBs) and the sample flow rate. A dosage of 60 µg IMBs consistently achieved a CCE of over 90%, making it the optimal dosage considering both experimental cost and efficiency (Figure S4). For the sample flow rate, 2.0 mL/h was found to be optimal, yielding a high capture efficiency of 90.98% ± 3.08% for HK2 cells, compared to only 4.17% ± 0.95% for CCRF‐CEM cells (Figure S5), while increasing the flow rate to 4.0 mL/h significantly reduced CCE to 41.93 %± 2.19% due to decreased interaction time between cells and antibodies. To evaluate the stability of the immunomagnetic‐bead (IMB) coating inside the RENAL‐Chip, 1.0 mL PBS was perfused at 2 mL/h. Microscopic bead counts showed that > 95% of the IMBs remained, confirming the excellent stability of bead coating (Figure S6). To further evaluate the chip's performance in capturing rare cells, 20 and 100 model cells were spiked into PBS buffer. The chip achieved a capture efficiency of 91.67% ± 3.06% for HK2 cells, while the non‐specific adsorption efficiency for CCRF‐CEM cells was only 4.27% ± 0.33% (Figure 2A). Additionally, the CCE was tested in real samples by spiking cells into whole blood. Linear analysis showed a strong correlation between the cell input numbers of HK2 or CCRF‐CEM cells and CCE, with slopes of 0.86 (R^2^ = 0.9982) and 0.054 (R^2^ = 0.9378), respectively (Figure 2B). These results demonstrate the chip's high efficiency in capturing and recovering rare cells. Furthermore, the capture performance of RENAL‐Chip in blood was compared with that of traditional HB‐Chip decorating with EpCAM antibodies and magnetic bead‐based sorting (MACS) methods. As shown in Figure 2C, the cell capture efficiency of the RENAL‐Chip was 84.40% ± 1.87%, while those of the HB‐Chip and MACS methods were only 74.20% ± 0.74% and 13.15% ± 0.49%, respectively. These results demonstrated that the magnetic reversible microfluidic interface exhibits superior CCE in blood samples.

The non‐destructive release and efficient recovery of CPCs are crucial for subsequent downstream analysis. Notably, the cell release efficiency was found to be 95.50% ± 0.71%, and further calculations showed that the recovery rate was 81.18% ± 3.04% (Figure 2D,E). Additionally, the purity of released HK2 cells from the chip was calculated to be 30.52% ± 0.42%. Given the presence of IMBs attached to the cell surface, magnetic separation was employed to further purify the cells, resulting in a cell purity of 77.14% ± 2.37% (Figure 2F). The impact of the chip on cell viability was assessed using Calcein‐AM/PI staining. The results revealed a high proportion of viable cells, reaching 96.04% ± 1.40%, while the proportion of dead cells was only 3.96% ± 1.40%, indicating minimal influence of the chip on cell viability (Figure 2G). Subsequently, the released HK2 cells were transferred to a culture dish for cultivation. It was observed that the cells maintained their integrity and exhibited normal growth, demonstrating their potential for re‐culture and downstream analysis (Figure 2H).

### 2.3 Identification and Quantification of CPCs for Renal Transplant Rejection Monitoring

**FIGURE 3:**
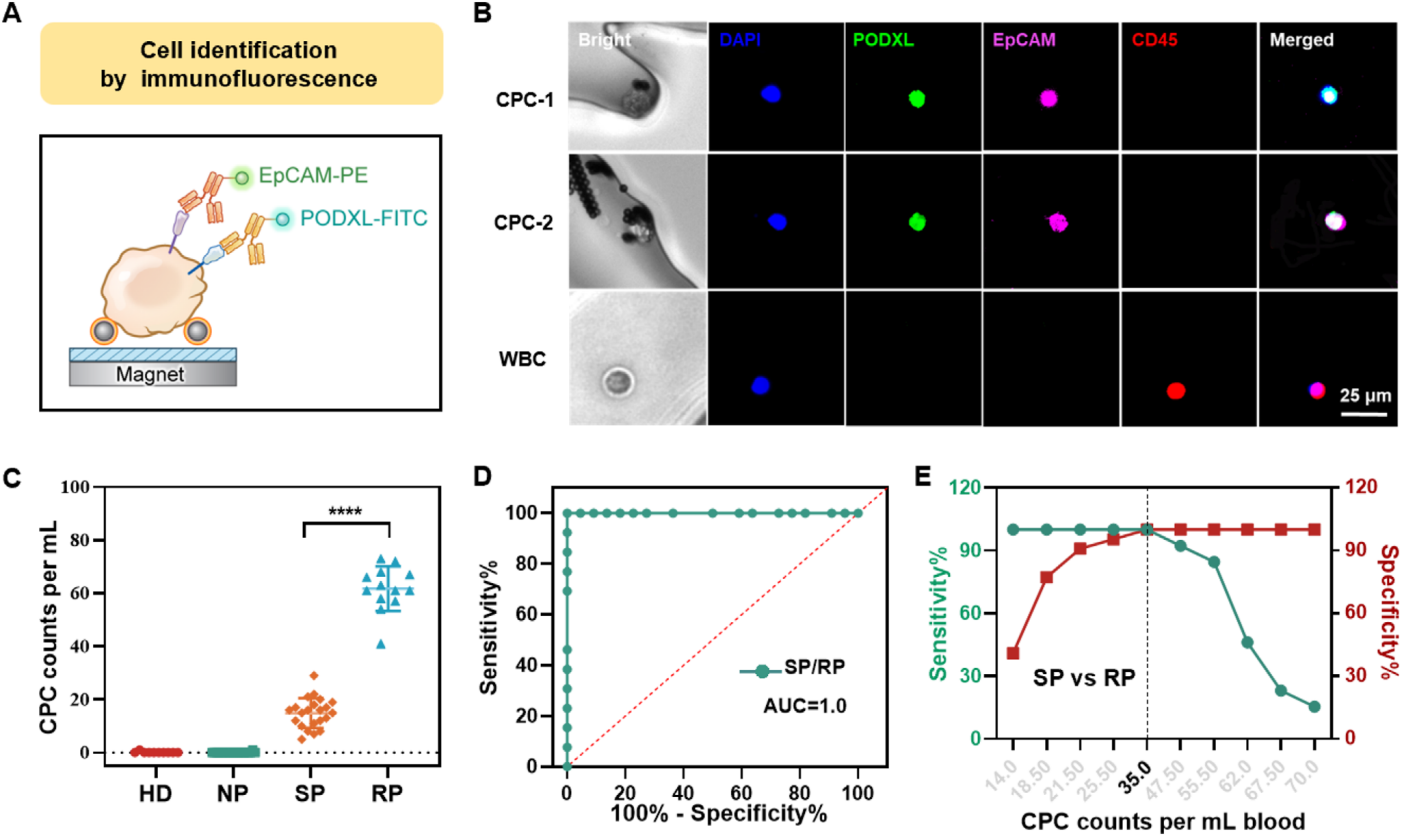
Identification and quantification of CPCs in clinical samples. (A) Schematic diagram showing cell identification by immunofluorescence. (B) Representative immunofluorescence images of CPCs and WBCs, scale bar = 25 µm. (C) Enumeration of CPCs isolated from clinical peripheral blood samples (^****^ indicates a *p*‐value <0.0001). (D) ROC analysis of CPC counts between the SP and RP groups. (E) Sensitivity and specificity curves of CPCs between the SP and RP groups.

To verify the presence of CPCs in the peripheral blood of renal transplant recipients experiencing rejection, we collected peripheral blood samples from 65 volunteers, including 10 healthy donors (HD), 20 nephritis patients (NP, without renal transplantation), and 35 renal transplant recipients (22 with a stable state (SP) and 13 with transplant rejection (RP)), and employed RENAL‐Chip for capturing and identifying CPCs in peripheral blood. Briefly, one milliliter of peripheral blood was injected into the chip for CPC capture, followed by immunofluorescence staining for cell identification. PODXL+/EpCAM+/CD45‐ cells were enumerated as CPCs, while CD45+/PODXL‐/EpCAM‐ were regarded as white blood cells (WBCs) (Figure 3A,B). As shown in Figure 3C, in the cohort of 35 renal transplant recipients, 8 to 73 CPCs per milliliter of blood were captured and identified. In contrast, non‐transplant individuals, including NP and HD, displayed only 0 to 1 CPC per milliliter of blood. A significant difference in CPC numbers was observed between SP and RP groups (*p* <0.0001). Receiver operating characteristic (ROC) curve analysis was applied to assess the sensitivity and specificity of this method. The area under the ROC curve (AUC) reached 1.0, indicating that CPC count is a good indicator for the prediction of renal transplant rejection (Figure 3D). According to the sensitivity and specificity curve, a CPC count of 35.0 cells/mL would be the diagnostic cutoff value to distinguish between the SP and RP groups (Figure 3E). Notably, the renal transplant rejection was confirmed by histopathological analysis of biopsies (Figure S7). Collectively, these findings confirm the presence of CPCs in the peripheral blood of renal transplant recipients undergoing rejection, highlighting their specificity to this condition.

### 2.4 Identification of CPC Origin Through Chromosome Analysis

**FIGURE 4:**
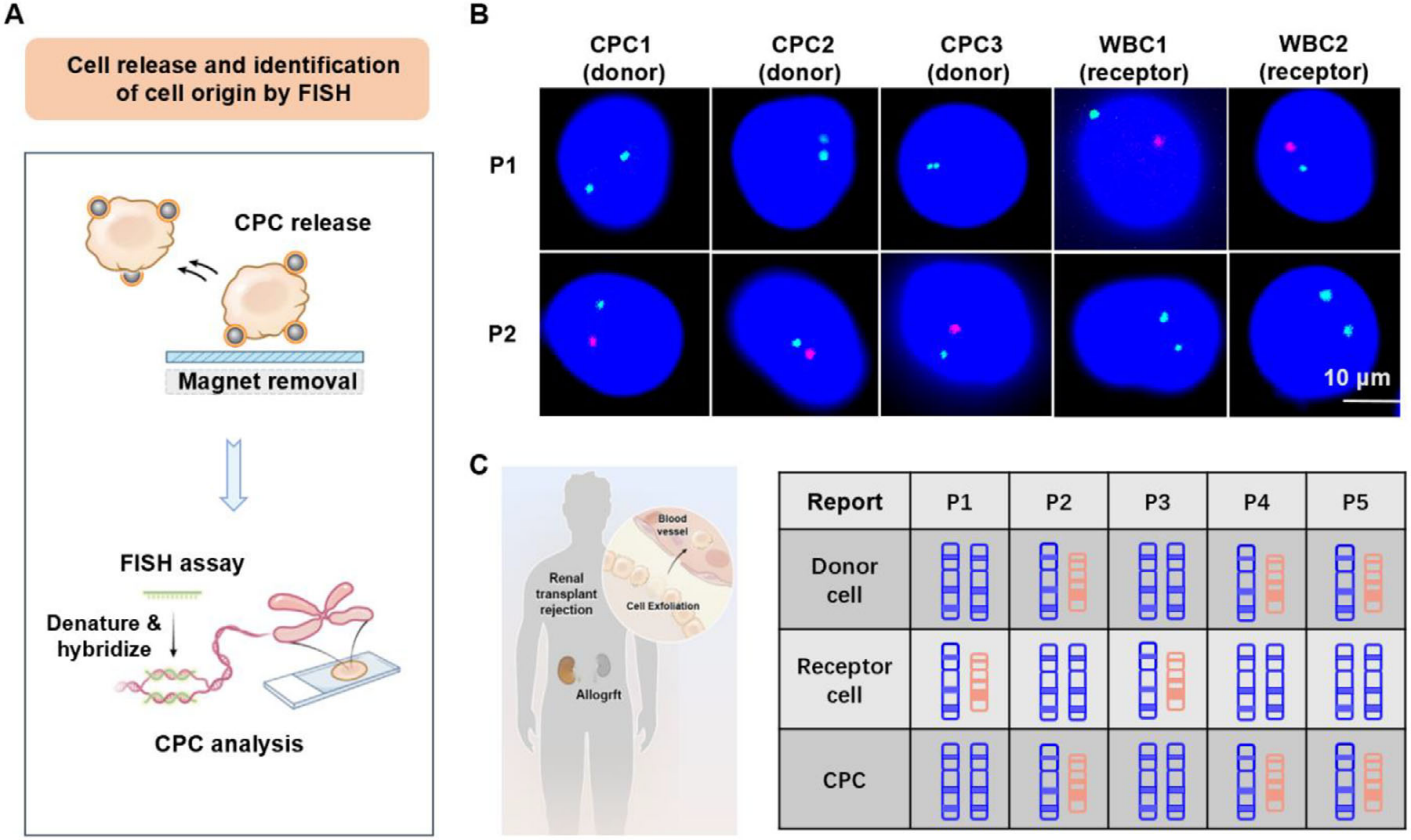
Identification of CPC origin by FISH. (A) Illustration of the cell retrieval and FISH procedure for chromosome analysis of released CPCs. (B) FISH analysis detecting the XY chromosomes in CPCs from renal transplant recipients (green for X, red for Y), scale bar = 10 µm. (C) FISH analysis report for renal transplant recipients. P1 and P3: recipient is male, donor is female; P2, P4, and P5: recipient is female, donor is male (orange for Y, blue for X).

To verify that the CPCs captured are derived from the donor kidney, we examined the XY chromosomes in CPCs obtained from gender‐mismatched donor‐recipient pairs. We collected peripheral blood samples from five patients who had undergone gender‐mismatched renal transplants. After capturing and recovering CPCs using the chip, we performed FISH analysis to determine the XY chromosome status of the CPCs (Figure 4A). In male recipients (P1 and P3), CPCs were found to express only the X chromosome, while WBCs expressed both X and Y chromosomes. In contrast, in female recipients (P2, P4, and P5), CPCs expressed both X and Y chromosomes, whereas WBCs expressed only the X chromosome (Figure 4B,C). These findings indicate that the identified CPCs originate from the renal transplant donors, thereby demonstrating the accuracy of this method.

### 2.5 scRNA‐Seq Reveals the Role of Immunity in Renal Transplant Rejection

**FIGURE 5:**
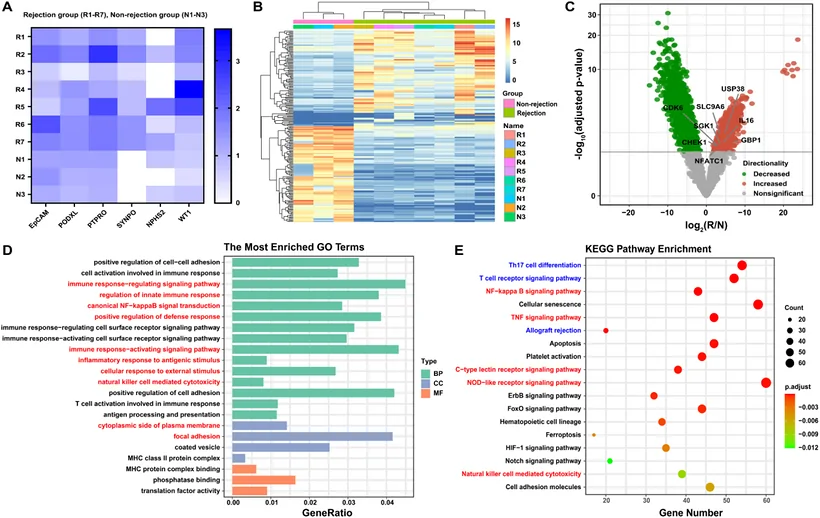
scRNA‐seq of isolated CPCs reveals the role of immunity in renal transplant rejection. (A) Marker genes detected in single CPCs, R denotes rejection CPCs, while N denotes non‐rejection CPCs. Expression levels are reported as log_10_ (n + 1), where n is the raw transcript count. (B) Heatmap depicting the expression of representative signature genes in CPCs from rejection and non‐rejection groups. (C) Volcano plot highlighting differentially expressed genes between rejection and non‐rejection groups in CPCs. Significantly upregulated or downregulated genes (|fold change| > 1 and padj < 0.05) are colored in red or blue, respectively. (D) GO analysis highlighting significant biological processes (BPs) and molecular functions (MFs). The GO terms highlighted in red are associated with innate immunity. (E) KEGG pathway enrichment analysis of isolated CPCs, highlighting the role of immunity in renal transplant rejection. Pathways highlighted in red are associated with innate immunity, whereas those highlighted in blue are related to adaptive immunity.

Thanks to its ability to efficiently and specifically enrich and recover CPCs from whole blood, RENAL‐Chip makes single‐cell sequencing and downstream analysis of these rare cells feasible. Historically, research on renal transplant rejection has predominantly focused on adaptive immunity. However, innate immunity has been increasingly recognized as a critical participant in transplantation in recent years. To elucidate the role of immunity in renal transplant rejection and the mechanism behind the increased number of CPCs, we conducted scRNA‐seq on CPCs from patients with and without renal transplant rejection. We manually picked seven CPCs from rejection patients and three CPCs from non‐rejection patients under a microscope, and prepared single‐cell RNA libraries according to the Smart‐seq2 protocol. Marker gene expression confirmed the podocyte identity of the isolated cells (Figure 5A), with their biological significance and functions detailed in Table S1. Hierarchical clustering revealed significant transcriptomic differences not only between rejection and non‐rejection groups but also among individual CPCs, underscoring the importance of single‐cell sequencing (Figure 5B). Compared with the SP group, the RP group exhibited significantly higher expression in CPCs of podocyte‐injury genes (CHEK1, GBP1, CDK6, SLC9A6, USP38) and immune‐activation genes (CDK6, IL16, NFATC1, SGK1), indicating that these two pathways jointly drive the initiation and progression of renal transplant rejection, ultimately resulting in chronic graft loss (Figure 5C). A summary of these gene functions and their potential implications for this study is provided in Tables S2 and S3.

Using the DAVID tool [21], we performed an in‐depth analysis of differentially expressed genes (DEGs) and assessed their biological processes (BPs), cellular components (CCs), and molecular functions (MFs). As shown in Figure 5D, several significant BPs and MFs were identified through GO analysis. Results showed that DEGs were predominantly enriched in the NF‐κB‐mediated innate/adaptive immune signaling pathway and extended into antigen presentation and cell adhesion/migration processes, indicating that immune system dysfunction is a key factor in renal transplant rejection. Particularly, the significant enrichment of pathways associated with innate immune responses, such as the regulation of innate immune response, canonical NF‐kappaB signal transduction, positive regulation of defense response, and inflammatory response to antigenic stimulus, further confirms that the activation of the innate immune system is a major contributor to transplant rejection. KEGG pathway enrichment analysis also revealed significant enrichment in innate immune pathways, including the C‐type lectin receptor signaling pathway, NOD‐like receptor signaling pathway, NF‐kappa B signaling pathway, TNF signaling pathway, and Natural killer cell mediated cytotoxicity (Figure 5E). This underscores the critical role of innate immunity in the rejection process. Moreover, enrichment was also noted in adaptive immune pathways, such as allograft rejection, as well as in Th17 cell differentiation and the T cell receptor signaling pathway. The results of the gene set enrichment analysis (GSEA, Figure S8) are consistent with the findings from the KEGG analysis. These findings illustrate the cooperative role of innate and adaptive immunity in renal transplant rejection.

## 3 Conclusions

The introduction of this magnetic reversible microfluidic interface for capturing and analyzing CPCs marks a significant leap forward in the non‐invasive diagnosis of renal transplant rejection. This research not only establishes CPCs as a robust biomarker for immune rejection monitoring but also offers valuable insights into the immunological mechanisms underlying this process. Compared to traditional diagnostic methods like renal biopsy, which are invasive and can cause patient discomfort and complications, our microfluidic interface provides a non‐invasive, more efficient, and reliable alternative. It achieves high capture efficiencies for targeted cells, with minimal non‐specific binding for non‐targeted cells in both PBS buffer and whole blood, highlighting the method's reliability and reproducibility. The non‐destructive release and efficient recovery of CPCs are vital for downstream analysis. Our results show that captured cells can be released with high efficiency, while maintaining high viability and purity. This ensures that the captured cells are suitable for downstream molecular and functional assays, especially scRNA‐seq, providing the high‐resolution data required to dissect the cellular and molecular drivers of renal‐allograft rejection and to guide patient‐specific therapies. The specificity is further validated by the identification of CPCs originating from the transplanted kidney using FISH assay. The detection of XY chromosomes in CPCs from gender‐mismatched donor‐recipient pairs confirms that these cells are indeed shed from the transplanted organ, providing direct evidence of renal cell shedding during immune rejection.

scRNA‐seq analysis of CPCs from patients with and without renal transplant rejection reveals significant differences in gene expression profiles. The upregulation of genes associated with podocyte injury (CHEK1, GBP1, CDK6, SLC9A6, USP38) and immune activation (CDK6, IL16, NFATC1, SGK1), underscores their synergistic role in driving renal transplant rejection. The enrichment of pathways related to C‐type lectin receptor signaling pathway, NOD‐like receptor signaling pathway, NF‐kappa B signaling pathway, TNF signaling pathway, and Natural killer cell mediated cytotoxicity highlights the involvement in innate immunity in renal transplant rejection. The enrichment of the HIF‐1 signaling pathway suggests potential tissue damage due to hypoxia, which may accelerate acute rejection responses. The TNF signaling pathway, a critical regulator of innate immune responses and inflammation, is activated by the binding of TNF‐α to its receptors (such as TNFR1 and TNFR2), thereby initiating downstream NF‐κB signaling. Overproduction or dysregulation of TNF signaling pathway members can activate the NF‐κB pathway, which is crucial for regulating both innate and adaptive immune responses as well as inflammatory processes. However, sustained activation or dysregulation may lead to inflammatory damage and the shedding of epithelial cells. These findings suggest that the activation of the innate and adaptive immune system may trigger a cascade of events leading to tissue damage and graft failure.

In conclusion, this study presents a novel, non‐invasive approach for the diagnosis of renal transplant rejection by leveraging the unique properties of CPCs. The combination of RENAL‐Chip with scRNA‐seq provides a powerful tool for monitoring transplant rejection and understanding the underlying immunological mechanisms. However, several limitations should be acknowledged. First, the low abundance of CPCs in clinical samples may constrain the detection sensitivity and overall diagnostic yield. Second, the high cost associated with single‐cell sequencing presents a barrier to widespread clinical implementation. Third, the feasibility of early diagnosis for renal transplant rejection remains to be validated through larger‐scale prospective studies. Addressing these challenges, future work should focus on enriching CPC capture efficiency to improve detection rates, developing cost‐effective sequencing strategies, and validating this approach in larger cohorts to enable real‐time monitoring and personalized treatment strategies. This technology holds promise for transforming post‐transplant care, enabling timely interventions to mitigate the risk of graft failure and improve patient outcomes.

## 4 Experimental Section

### 4.1 Reagents and Materials

PBS (20×), bovine serum albumin (BSA), 4% paraformaldehyde (PFA), anti‐IgG (PE), and goat serum (GS) were sourced from Sangon Biotech Co., Ltd. (Shanghai, China). SU‐8 photoresist, polydimethylsiloxane (PDMS; Sylgard 184, Dow Corning), and silicon wafers (4 inches) were provided by Suzhou Cchip Scientific Instrument CO., Ltd. (Suzhou, China). Dynabeads‐M280‐Streptavidin (2.8 µm), 4,6‐diamidino‐2‐phenylindole (DAPI), and Calcein AM were obtained from Thermo Fisher Scientific (Massachusetts, USA). Biotinylated anti‐EpCAM antibody (BAF960) was obtained from R&D Systems (Minneapolis, USA). Anti‐EpCAM‐PE (Clone: VU‐1D9) and live/dead cell detection kit were purchased from Sigma–Aldrich (Shanghai, China). Alexa Fluor 488‐conjugated anti‐PODXL (Clone: EPR9518) and anti‐CD45‐APC (Clone: MEM‐28) were sourced from Abcam (Cambridge, UK). X/Y Chromosome fluorescence in situ hybridization (FISH) kits were provided by Guangzhou Anbiping Medical Technology Co., Ltd. (Guangzhou, China). Anti‐IgG‐PE (Clone: MOPC‐21) was provided by BioLegend, Inc. (San Diego, USA).

### 4.2 Flow Cytometry Analysis

To evaluate the expression levels of the epithelial cell adhesion molecule (EpCAM) in HK2 (human renal cell lines) and CCRF‐CEM (human acute lymphoblastic leukemia T lymphocytes), approximately 2×10^5^ cells were incubated with either 5 µL of anti‐EpCAM (PE) or anti‐IgG (PE) in 50 µL of 0.05% BSA (in PBS) for 1 h at room temperature. Following two washes with PBS, the cells were resuspended in 200 µL of PBS. A total of 10 000 events were acquired and analyzed using a Beckman Cytoflex FACS Verse flow cytometer.

### 4.3 Chip Preparation

The chip fabrication process was adapted from our previous work [22]. Briefly, the PDMS prepolymer (mixed in a 10:1 ratio of PDMS base to curing agent) was poured into a herringbone‐patterned mold and then heated to solidify. Once solidified, the PDMS chip was carefully peeled off and punched to create the necessary openings. To prepare the immunomagnetic beads (IMBs), Dynabeads‐M280‐Streptavidin (SA‐MBs) were incubated with biotinylated anti‐EpCAM antibodies (weight ratio of biotinylated anti‐EpCAM to SA‐MBs = 1:100) in a solution of 3% BSA (in PBS) for 30 min at 25 °C. After two washes with PBS using magnetic separation, the IMBs were resuspended in 20 µL of 3% BSA (in PBS). Next, a 20 µL solution of the prepared IMBs was introduced into the PDMS chip using an Eppendorf pipette. Finally, the functionalized chip underwent a 20‐min magnetic attraction process to ensure that the IMBs were fully attracted to the chip's substrate.

The local antibody surface density was calculated as the amount of antibody on the SA‐MB divided by the total surface area of the SA‐MB. The mass of unreacted antibody was quantified by bead‐ELISA. Briefly, equal numbers of SA‐MBs were incubated with increasing masses of biotinylated antibody for 15 min. After removal of unbound antibody, the beads were treated with a fluorescent secondary antibody for 10 min, washed, and analyzed by flow cytometry to obtain the mean fluorescence intensity (MFI) of the bead population. The MFI was further used to construct a standard curve. The mass of reacted antibody was calculated as m = m_1_ – m_2_, where m_1_ is the initial mass of antibody added, and m_2_ is the mass of unreacted antibody recovered. The corresponding number of antibody molecules bound to the beads was then determined using N_ab_ = (m / MW) × NA, where MW is the molecular weight of the antibody (150 kDa) and NA is Avogadro's constant (6.02 × 10^2^ ^3^). Finally, the local antibody surface density was computed as C = N_ab_ / (N_beads_ × πd^2^), where N_beads_ (the number of beads) is obtained by optical microscopy and d is the diameter of SA‐MB (2.8 µm).

### 4.4 Evaluation of Chip Performance

To evaluate the capture performance of the chip, Calcein‐AM‐stained HK2 and CCRF‐CEM cells were resuspended in PBS or healthy human peripheral blood. These cell suspensions were introduced into the chip at a flow rate of 2.0 mL/h using a Harvard Apparatus syringe pump to facilitate cell capture. The number of cells introduced and captured by the chip was counted using a Nikon fluorescence microscope. Cell capture efficiency was defined as the percentage of the number of captured cells relative to the number of introduced cells. After capturing HK2 cells on the chip, the magnet was removed, and 1.5 mL of a 5% BSA solution (in PBS) was pumped into the chip to release the captured cells. The solution containing the released cells was collected in a culture dish. The number of released HK2 cells was counted under a fluorescence microscope to evaluate the cell release efficiency, which was defined as the percentage of the number of released cells relative to the number of captured cells. Additionally, the released cells were stained with 1× Hoechst 33342 to assess their viability and purity. One hundred Calcein‐AM‐labeled HK2 cells were spiked into 1 mL of peripheral blood, captured by the RENAL‐Chip, and subsequently stained with Hoechst 33342. Calcein‐AM‐positive cells were identified as HK2 cells, while Hoechst‐positive cells represented the total cell population. The ratio of these two populations was defined as the target cell purity. Since HK2 cells were conjugated with immunomagnetic beads, whereas non‐specific cells remained unlabeled, magnetic separation further enhanced the purity of the HK2 cells.

### 4.5 Evaluation of Cell Viability

To assess the impact of the chip on cell viability, we utilized a live/dead cell detection kit. After releasing and collecting the cells captured by the chip, we stained them with PI (propidium iodide) and Calcein‐AM for 15 min at room temperature. Cell viability was evaluated based on fluorescence signals, where red fluorescence indicated dead cells and green fluorescence represented live cells. Additionally, we examined the in vitro culture capabilities of the released HK2 cells. The released cells were placed in a culture medium and incubated at 37 °C in an atmosphere of 5% CO_2_. Microscopic images of cell growth were captured at 0‐, 4‐, 8‐, and 24‐h post‐incubation to monitor their growth status.

### 4.6 CPC Identification and Enumeration Assay

Peripheral blood samples were collected in compliance with the protocols approved by the Human Research Ethics Committee at the Xiang An Affiliated Hospital of Xiamen University (Project ID: XMHLL2021028). Informed consent was obtained from all participants prior to the blood draw. Detailed donor information is provided in Table S4. Peripheral blood samples were collected using anticoagulant tubes containing EDTA and stored at 4°C. To assess the clinical applicability of this method, a 1 mL peripheral blood sample was introduced into the chip for cell capture at a flow rate of 2.0 mL/h. To immobilize the captured cells, a 4% paraformaldehyde (PFA) solution was introduced and incubated at room temperature for 15 min. After washing with PBS buffer, the cells were stained for 1 h with a cocktail of antibodies, including 5 µg/mL anti‐EpCAM‐PE, 5 µg/mL Alexa Fluor 488‐conjugated anti‐PODXL, and 5 µg/mL anti‐CD45‐APC. Subsequently, 2 µg/mL DAPI (in PBS) was added to stain the cell nuclei. Finally, the captured cells were imaged using a Nikon Ti‐U fluorescence microscope for identification.

### 4.7 Fluorescence In Situ Hybridization (FISH) Assay

FISH staining of the released cells was performed according to the protocol provided with the X/Y chromosome FISH kit. In summary, the released cells were fixed in 200 µL of fixation solution (prepared by mixing glacial acetic acid and methanol in a 1:3 ratio) at room temperature for 15 min, followed by incubation at −20 °C for 30 min. The cell suspension was then centrifuged at 2000 rpm for 5 min, and the supernatant was removed. Approximately 5 µL of the cell mixture was applied to a poly‐lysine‐coated slide to prepare cell smears, and this process was repeated until all solutions were applied. The slides were incubated overnight in an oven at 65 °C, followed by sequential washes in 2× SSC buffer and ethanol solutions at concentrations of 70%, 90%, and 100%, each for 2 min. After air‐drying the smears, the cells were incubated with the FISH probe mixture, denatured at 78 °C for 2 min, and then hybridized with the X/Y chromosomes in a dark chamber at 37 °C for approximately 48 h. The slides were subsequently immersed in a 0.3% NP‐40/SSC solution (72 °C, 2 min), a 0.1% NP‐40/2× SSC solution (30 s), and ethanol solutions at concentrations of 70%, 90%, and 100% (2 min each), before being dried in the dark. Finally, the nuclei were stained with 10 µL of nuclear dye solution, covered with a coverslip, and observed under a Nikon Ni‐U upright fluorescence microscope.

### 4.8 CPC Retrieval and Transcriptome Amplification

After capturing CPCs from peripheral blood, the cells were released and stained with a fluorescent antibody mixture at room temperature for 1 h. Subsequently, the nuclei were stained with 1× Hoechst for 10 min. Single‐cell transcriptome amplification was performed following the Smart‐seq2 protocol by Picelli et al., [23]. CPCs were picked using micromanipulation and immediately transferred to a 200‐µL thin‐walled tube containing lysis buffer (comprising 0.1% Triton X‐100, 5 U/µL Takara Recombinant RNase Inhibitor, 2.5 mm dNTPs, and 2.5 µM oligo‐dT). The cells were lysed at 72 °C for 3 min to ensure sufficient cell lysis. Following lysis, 5.7 µL of reverse transcription (RT) reaction solution (including 10 U/µL Takara Recombinant RNase Inhibitor, 10 U/µL Thermo Fisher Superscript II reverse transcriptase, 1 µm TSO primer, 1 m betaine, 1× RT buffer, 6 mm MgCl_2_, and 5 mm DTT) was added immediately. The reaction was conducted at 42 °C for 90 min, followed by 15 min at 70 °C to inactivate the RT enzyme. Lastly, 15 µL of PCR mix (comprising 12.5 µL 2× KAPA HiFi Mix, 0.25 µL 10 µm IS PCR primer, and 2.25 µL DEPC water) was added, and cDNA was amplified for 25 cycles in a PCR thermocycler to prepare single EC transcriptome samples. After amplification, the cDNA product was purified using VAHTS DNA Clean Beads at a 0.6 (V/V) ratio and eluted with 20 µL of DEPC‐treated water to obtain cDNA fragments of approximately 700–1000 bp in length. The purified cDNA was then quantified using the Qubit reagent kit. Finally, 5 ng of cDNA was used to prepare sequencing libraries for the Illumina NovaSeq X‐Series Sequencer with the DNA Library Prep Kit (Vazyme, TD502) according to the manufacturer's instructions.

### 4.9 Transcriptome Data Analysis

The obtained paired‐end sequencing data were initially processed with Trim Galore (https://www.bioinformatics.babraham.ac.uk/projects/trim_galore/, version 0.6.10) to remove adapters. Following adapter removal, the data were aligned to the human genome (hg38) using STAR (version 2.7.10b), resulting in BAM files sorted by genomic coordinates. Quantification of the aligned BAM files was performed using FeatureCounts (version 2.0.6) [24]. Differential expression analysis was conducted on the resulting expression matrix using DESeq2 (version 1.36.0) [25]. After establishing the appropriate sample grouping, a DESeqDataSet (DDS) object was constructed, and dispersion was estimated using the DESeq function to derive the differential expression results. The thresholds for selecting differentially expressed genes during enrichment analysis were set as follows: log2FC: log2(10), *p*‐value: 0.001, and adjusted *p*‐value (padj): 0.001. The heatmap was generated based on normalized counts from the featureCounts program (|fold change| > 1 and padj <0.05) using the heatmap function (version 1.0.12). Gene Ontology (GO) and Kyoto Encyclopedia of Genes and Genomes (KEGG) analyses for differentially expressed genes (|fold change| > 1 and padj <0.05) were performed utilizing the Database for Annotation, Visualization, and Integrated Discovery (DAVID) bioinformatics resources.

## Author Contributions

C.S., H.Z., and C.Y. conceived the study. J.S. conducted most of the experiments with help from Z.Z., Y.Z., Y.Z., X.Y., and J.Z. M.Z. performed most of the bioinformatic analysis. P.L. helped with the bioinformatic analysis. Y.L. analyzed the data and wrote the manuscript with inputs from all authors. C.S., H.Z., and C.Y. supervised all aspects of this study.

## Funding

This research was supported by the National Natural Science Foundation of China (No. 22274066, No. 82472737), the National Key R&D Program of China (No. 2024YFF1206600), Xiamen Ocean and Fisheries Development Special Funds Program (No. 22CZP006HJ03), and Scientific Research Foundation of State Key Laboratory of Vaccines for Infectious Diseases, Xiang An Biomedicine Laboratory (No. 2023XAKJ0100080)

## Conflicts of Interest

The authors declare no conflicts of interest.

## Supporting information




**Supporting File**: advs74908‐sup‐0001‐SuppMat.docx.

## Data Availability

The data that support the findings of this study are available from the corresponding author upon reasonable request.
