# Supplementary material for: RENAL‐CHIP: Rejection Evaluation via Non‐Invasive Analysis of Circulating Podocytes With Herringbone‐Chip Isolation Platform
Source: Adv Sci (Weinh). 2026 Mar 18;13(31):e20426. doi: 10.1002/advs.202520426 (PMC13587950; doi:10.1002/advs.202520426)
Supplement: Supplementary file 1 [file ADVS-13-e20426-s001.docx]

Supporting Information

RENAL-CHIP: Rejection Evaluation via Non-invasive Analysis of Circulating Podocytes with Herringbone-chip Isolation Platform

Juan Song,^#^ Yilong Liu,^#^ Zeyuan Zheng,^#^ Ming Zhu, Yuning Zou, Pei Liu, Yue Zheng, Xiyuan Yu, Jianzhong Zheng, Chen Shao,* Huimin Zhang,* and Chaoyong Yang*

#Juan Song, Yilong Liu, and Zeyuan Zheng contributed equally to this work.

**Figure S1.** EpCAM^+^ / PODXL^+^ podocytes were identified by immunofluorescence in different renal allograft rejection biopsy tissues. Scale bar = 10 μm.

**Figure S2.** Characterization of the magnetic reversible microfluidic interface. (A) Dimensions of the magnetic reversible microfluidic interface. (B) Pre-assembly characterization of the chip. (C) Post-assembly characterization of the chip with IMB-PE. (D) Characterization of the chip after IMB-PE release. Scale bar = 25 μm.

**Figure S3.** Flow cytometry analysis to characterize the EpCAM expression in HK2 and CEM cells. 50,000 cells of each cell line were stained with anti-IgG-PE and anti-EpCAM-PE, respectively.

**Figure S4.** Optimization of IMB dosage for RENAL-Chip modification. To optimize the immunomagnetic-bead loading amount in the HB-Chip, we loaded increasing amounts of beads and quantified the capture efficiency of HK-2 cells, identifying 50 µg as the optimal bead load. Data are presented as mean ± SD from three independent experiments.

**Figure S5.** Optimization of flow rate of RENAL-Chip for cell capture. To optimize the flow rate for RENAL-Chip sample processing, we determined the capture efficiencies of HK-2 and CCRF-CEM cells in PBS buffer across a range of flow rates. Balancing sample throughput and cell capture efficiency, we identified 2 mL/h as the optimal cell-capture flow rate. Data are presented as mean ± SD from three independent experiments.

**Figure S6.** Characterization of the chip stability. Typical microscopy images show the IMB loading on the RENAL-Chip before (A) and after (B) 1 mL PBS perfusion. Subsequent bead counts revealed that >95% of the beads were retained, demonstrating excellent on-chip stability of the immunomagnetic-bead coating.

**Figure S7.** Routine staining of the renal biopsy tissue, including HE, PAS, PASM, and Masson. Representative images confirm immune-mediated rejection of the renal allograft. This data was provided by the Xiang’an Affiliated Hospital of Xiamen University.

**Figure S8.** Gene-set enrichment analysis (GSEA) of CPCs. Gene-set enrichment analysis (GSEA) of distinct pathways related to renal-allograft rejection was performed to compare the transcriptomes of rejection versus non-rejection groups.

**Table S1.** Podocyte marker genes and their biological significance and function

| Gene symbol | Encoded protein | Biological significance and function |
| --- | --- | --- |
| *EpCAM* | Epithelial cell adhesion molecule | A classic epithelial cell marker mediating intercellular adhesion. Upon severe injury, dedifferentiated podocytes aberrantly express EpCAM, indicating active disease. |
| *PODXL* | Podocalyxin | Strongly negatively charged sialic acid glycoproteins on the podocyte surface prevent mutual podocyte adhesion and adhesion to the glomerular basement membrane through electrostatic repulsion, thereby maintaining foot process separation and the open architecture of the slit diaphragm. |
| *PTPRO* | Glomerular epithelial protein 1 | Located at the apex of podocyte foot processes, it participates in regulating cytoskeletal architecture, foot process morphology, and cell migration. |
| *SYNPO* | Synaptopodin | A specific marker of podocyte differentiation that links the plasma membrane to the actin cytoskeleton, stabilizes foot process architecture, and couples cytoskeletal dynamics with signal transduction. |
| *NPHS2* | Podocin | A key component of the slit diaphragm complex that recruits transmembrane proteins (e.g., Nephrin, TRPC6) to lipid raft microdomains and activates downstream signaling pathways (such as MAPK), thereby playing an essential role in maintaining filtration barrier integrity. |
| *WT1* | Wilms’ tumor 1 | A core transcription factor maintaining the terminally differentiated state of podocytes. It constitutively activates the expression of podocyte‑specific genes (e.g., NPHS2, PODXL) and suppresses dedifferentiation. |

**Table S2.** Podocyte injury-related genes, their functions and relevance to the study.

| Gene symbol | Biological significance and function | Podocyte injury mechanisms |
| --- | --- | --- |
| *CHEK1* | A core kinase of the DNA damage response that maintains genomic stability through G2/M cell cycle arrest, preventing damaged DNA from entering mitosis. | DNA damage accumulation → cell apoptosis |
| *GBP1* | An interferon-induced immune GTPase that mediates cell-autonomous immunity and antiviral responses by remodeling the cytoskeleton and inducing autophagy to eliminate intracellular pathogens. | IFN signaling / inflammasome hyperactivation |
| *CDK6* | A cyclin-dependent kinase that drives G1-S phase transition to promote cell proliferation, possesses transcriptional regulatory functions, and plays critical roles in cell cycle control, differentiation, and angiogenesis. | Cell cycle re-entry → mitotic failure |
| *SLC9A6* | An early endosome-localized sodium-hydrogen exchanger that maintains endosomal pH homeostasis and vesicular trafficking, thereby regulating neurotrophic signaling and synaptic plasticity. | Endosomal acidification / trafficking impairment |
| *USP38* | A deubiquitinase that regulates protein turnover, epigenetic modification, and signal transduction by stabilizing substrate proteins. | Protein stability imbalance / inflammatory signaling |

**Table S3.** Immune-activation genes genes, their functions and relevance to the study.

| Gene symbol | Biological significance and function | Underlying immune mechanisms |
| --- | --- | --- |
| *CDK6* | A cyclin-dependent kinase that drives the G1-to-S phase transition to promote cell proliferation, possesses transcriptional regulatory functions, and participates in cell differentiation and angiogenesis. | Upregulation of CDK6 in podocytes marks a dedifferentiated state, rendering these cells more susceptible to immune injury. |
| *IL16* | A pro-inflammatory cytokine that primarily chemoattracts CD4⁺ T cells, monocytes, and eosinophils, participating in immune responses, inflammatory reactions, and the pathogenesis of autoimmune diseases. | Injured podocytes upregulate IL16 secretion to directly recruit CD4⁺ T cells and macrophages into the glomerulus, thereby forming a local inflammatory microenvironment. |
| *NFATC1* | A calcium-dependent transcription factor that regulates T/B cell activation, differentiation, and cytokine (e.g., IL-2) expression; also implicated in osteoclastogenesis, cardiac hypertrophy, and tumorigenesis. | By regulating inflammation-related genes (e.g., COX-2, TNF-α), it enhances its own inflammatory phenotype |
| *SGK1* | A serum- and glucocorticoid-regulated kinase that critically modulates epithelial sodium channel (ENaC)-mediated ion homeostasis and blood pressure regulation, and participates in cell survival, proliferation, migration, and fibrosis. | Chronic activation promotes fibrosis and modulates NLRP3 inflammasome activity, thereby converting metabolic stress into chronic inflammation. |

**Table S4.** Detailed information of blood donors.

| Sample ID | Gender | Clinical diagnosis | CPC counts per mL blood |
| --- | --- | --- | --- |
| 1 | male | renal transplant rejection patient | 68 |
| 2 | female | renal transplant rejection patient | 61 |
| 3 | male | renal transplant rejection patient | 63 |
| 4 | male | renal transplant rejection patient | 72 |
| 5 | male | renal transplant rejection patient | 73 |
| 6 | male | renal transplant rejection patient | 67 |
| 7 | female | renal transplant rejection patient | 61 |
| 8 | male | renal transplant rejection patient | 61 |
| 9 | female | renal transplant rejection patient | 66 |
| 10 | female | renal transplant rejection patient | 58 |
| 11 | male | renal transplant rejection patient | 54 |
| 12 | female | renal transplant rejection patient | 57 |
| 13 | female | renal transplant rejection patient | 41 |
| 14 | male | stable allograft patient | 16 |
| 15 | female | stable allograft patient | 16 |
| 16 | female | stable allograft patient | 29 |
| 17 | female | stable allograft patient | 19 |
| 18 | male | stable allograft patient | 12 |
| 19 | female | stable allograft patient | 22 |
| 20 | male | stable allograft patient | 17 |
| 21 | male | stable allograft patient | 20 |
| 22 | female | stable allograft patient | 8 |
| 23 | female | stable allograft patient | 18 |
| 24 | male | stable allograft patient | 21 |
| 25 | male | stable allograft patient | 15 |
| 26 | female | stable allograft patient | 17 |
| 27 | male | stable allograft patient | 16 |
| 28 | female | stable allograft patient | 11 |
| 29 | male | stable allograft patient | 12 |
| 30 | male | stable allograft patient | 5 |
| 31 | male | stable allograft patient | 7 |
| 32 | female | stable allograft patient | 8 |
| 33 | female | stable allograft patient | 10 |
| 34 | female | stable allograft patient | 15 |
| 35 | female | stable allograft patient | 13 |
| 36 | male | nephritis patient | 0 |
| 37 | male | nephritis patient | 0 |
| 38 | female | nephritis patient | 0 |
| 39 | male | nephritis patient | 0 |
| 40 | female | nephritis patient | 0 |
| 41 | male | nephritis patient | 0 |
| 42 | female | nephritis patient | 0 |
| 43 | male | nephritis patient | 0 |
| 44 | male | nephritis patient | 0 |
| 45 | male | nephritis patient | 0 |
| 46 | male | nephritis patient | 0 |
| 47 | male | nephritis patient | 0 |
| 48 | male | nephritis patient | 0 |
| 49 | male | nephritis patient | 0 |
| 50 | male | nephritis patient | 0 |
| 51 | male | nephritis patient | 0 |
| 52 | male | nephritis patient | 0 |
| 53 | male | nephritis patient | 0 |
| 54 | male | nephritis patient | 0 |
| 55 | female | nephritis patient | 0 |
| 56 | male | healthy donor | 0 |
| 57 | male | healthy donor | 0 |
| 58 | male | healthy donor | 0 |
| 59 | male | healthy donor | 0 |
| 60 | male | healthy donor | 0 |
| 61 | male | healthy donor | 0 |
| 62 | male | healthy donor | 1 |
| 63 | male | healthy donor | 0 |
| 64 | male | healthy donor | 0 |
| 65 | male | healthy donor | 0 |
